# The morphologic correlation between vortex transformation and upper critical field line in opal-based nanocomposites

**DOI:** 10.1038/s41598-021-84343-1

**Published:** 2021-02-26

**Authors:** M. K. Lee, E. V. Charnaya, S. Mühlbauer, U. Jeng, L. J. Chang, Yu. A. Kumzerov

**Affiliations:** 1 MOST Instrument Center at NCKU, Tainan, 70101 Taiwan; 2 Department of Physics, National Cheng Kung University, Tainan, 70101 Taiwan; 3 Department of Physics, St. Petersburg State University, St. Petersburg, Petrodvorets 198504 Russia; 4 Heinz Maier-Leibnitz Zentrum (MLZ), Technische Universität München, Lichtenbergstrasse 1, 85748 Garching, Germany; 5 National Synchrotron Radiation Research Center, 101 Hsin-Ann Road, Hsinchu Science Park, Hsinchu, 30076 Taiwan; 6 A. F. Ioffe Physico-Technical Institute RAS, St. Petersburg, 194021 Russia

**Keywords:** Nanoparticles, Phase transitions and critical phenomena, Superconducting properties and materials

## Abstract

In this study, we investigate metallic nanocomposites to elucidate the properties of nanostructured conventional superconductors. Liquid tin, indium, and mercury are loaded into opal matrices by high pressure up to 10 kbar. The opal templates preserve the 3D dendritic morphology of confined superconducting metals to model a dendritic second phase with particular grain shape in bulk superconductors observed by a DualBeam microscope. We carry out measurements of the dc and ac magnetizations to study the superconducting phase diagrams, vortex dynamics, and impact of grain morphology in the opal composites. Besides, we apply the small-angle neutron scattering (SANS) to deny a regular vortex structure. The phase diagrams reveal an enhanced upper critical field *H*_*c*2_(0) and curvature crossover in the upper critical field line. We also calculate the vortex activation barriers *U*_*a*_ and observe a transformation in the vortex system. According to the field dependence of *U*_*a*_, the vortex structure transformation highly correlates with the curvature crossover in the upper critical field line. Our observations suggest that the similarity in the normalized phase diagrams and field dependences of *U*_*a*_ in the three nanocomposites is owing to their particular morphology of confinement.

## Introduction

Most superconductivity studies in recent decades aimed at investigating the physical properties of unconventional superconductors like heavy fermion superconductors^1^, high temperature superconductors^2,3^, borocarbides^4^, and iron-based superconductors^5^, owing to their potential high temperature and high current applications. However, even the phenomenology in unconventional superconductors remains highly disputable yet. This ambiguity largely arises from inhomogeneity in bulk superconductors, subsequently leading to introduction of different amounts of pinning centers into them. Accordingly, vortex dynamics impact greatly on superconducting properties for non-perfect superconductors.

The inability to clarify how granular and morphologic effects influence superconductivity poses a major obstacle for understanding the behavior of various kinds of superconductors. A particular signature of multiband superconductors, superconductors with an anisotropic Fermi surface, and of d-wave superconductors is the upturned or positive curvature of the upper critical field line^6,7^. Withal, the positive curvature appears as well in granular and nanostructured superconductors^8,9^. All these different mechanisms can cause the similar unconventional behavior, which often makes difficult to distinguish scenarios. Understanding the morphology effects on superconductors as a missing puzzle piece demands more efforts in the field of superconductivity and is vital to comprehend the specific features induced by inhomogeneity and grain shapes. With these points of view, studies of nanostructured single-element superconductors comprising tremendous pinning centers might help shed some light on how to distinguish the inherent properties of superconductors from granular and morphology effects at the nanoscale.

The superconductivity in zero-dimensional superconductors, one-dimensional superconductors, and two-dimensional superconductors differ significantly from each other and bulk behavior^10,11^. Most of all nanostructured superconductor studies mainly relate with quantum dots, strip, and film geometry instead of dendrites (owing to the fragility and difficulty in preserving dendritic morphology) which shape is possible for grains. Besides, nanocomposites such as porous glasses loaded with superconducting metals show some general properties like strongly enhanced critical field and critical current comparing to bulk materials. Loaded porous matrices always behaved as type-II dirty superconductors leading to unusual magnetic instabilities, too. The hierarchy of strong and weak Josephon links affected the temperature dependences of their magnetic features. With these fascinating behaviors, the community has devoted decades of studies to this field^9,12–15^. But, the role of morphology for the superconducting nano-inclusion was still unrevealed until now.

To recognize the influence of morphology on nanostructure superconductivity, we take nanoporous opal templates and embedded Sn, In, and Hg into pores. These metals are type-I superconductors in bulk. The opals consist of close-packed silica spheres^16^ with interconnected pores between them and keep loaded metals maintaining a dendritic geometry^17,18^. The insulating silica spheres ensure strong pinning while metallic inclusions themselves are homogeneous and provide weaker pinning. It is just opposite to the regular arrays of artificial pinning centers^11,19,20^. We take advantage of the nanocomposite structure to construct an ensemble of nanosized superconductors with particular morphology linked with each other. We apply dc and ac magnetization measurements at different temperatures and fields. We show that all our opal-based superconducting nanocomposites have similar phase diagrams with the enhanced upper critical fields *H*_*c*2_(0) and curvature crossover on the upper critical field line. The crossover field agrees with a bend on the field dependence of the activation barrier *U*_*a*_ which indicates a vortex transformation. Moreover, a common value of the reduced crossover field *H*_*cr*_/*H*_*c*2_(0) ~ 0.35 is found for the nanostructured superconductors under study. Our work demonstrates an unconventional behavior in conventional metallic superconductors with a particular grain morphology kept by an opal template.

## Results

### SEM micrograph

**Figure 1:**
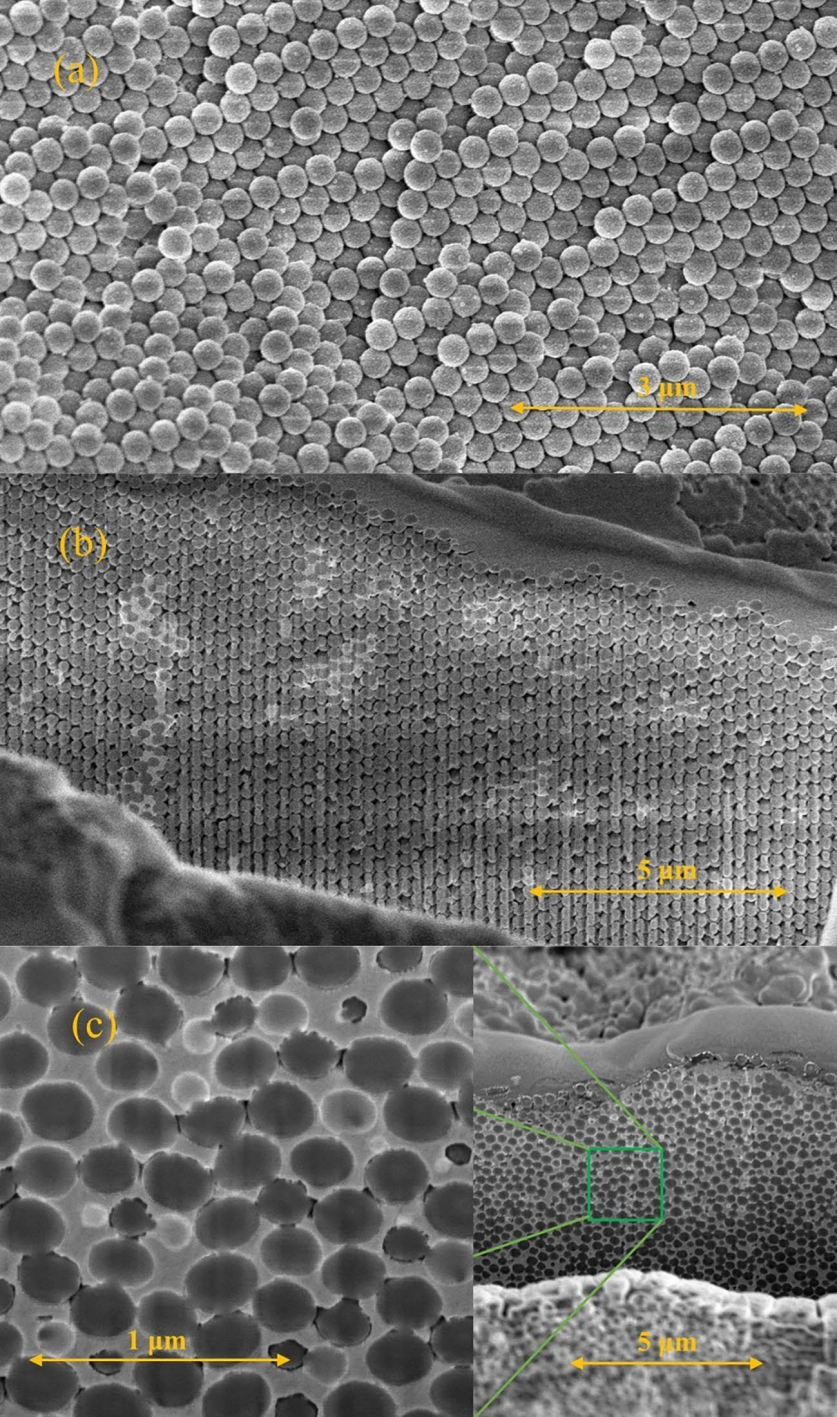
SEM images of opal nanocomposites with scales. (**a**) A micrograph for O-In on the surface, there is almost no any indium in pores indicating a very low filling factor near surface. The small particles on silica spheres are platinum (to improve SEM image). (**b**) The cross section of O-In milled by the Ga ion beam; pores are partially filled by indium. (**c**) The cross section images of O-Sn, all pores are almost filled by tin. On the left side, we show an enlarged photo.

We show the nanostructure of tin and indium (O-Sn and O-In) embedded into our opal template in Fig. 1a–c. The opal matrix maintains its superlattice structure after loading metals into pores. Near the surface of O-In, almost all pores are empty (Fig. 1a). The cross-section view of O-In reveals a ca. 15% of pores partially filled by indium. However, the average filling factor of O-In is ca. 60% (see "Methods"). The difference is owing to that the indium near the sample surface tends to flow out. For O-Sn, we choose the core to get a piece with the filling factor closed to 100%. The cross-section view indicates a filling factor above 90%. We take this particular piece to check whether the filling factor well above 60% influences the superconductivity strongly or not. Owing to the mismatch of alignment, the silica spheres show an *oval* shape. For the opal loaded with mercury (O-Hg), the mercury is exuding from pores when the FIB mills the cross-section. The picture is very similar to Fig. 1b with much less inclusion.

### dc magnetization

**Figure 2:**
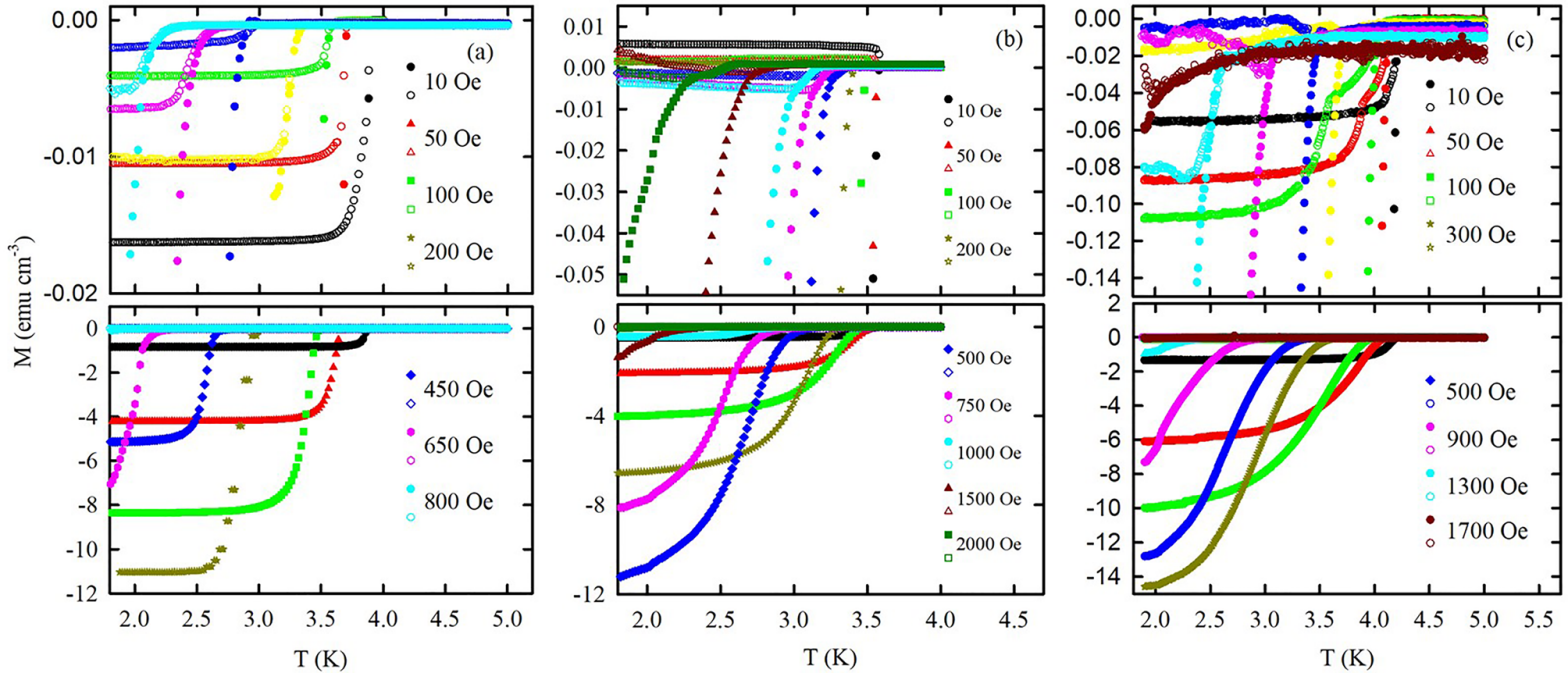
Temperature dependences of dc magnetization under ZFC (closed symbols) and FC (open symbols) protocols at different external magnetic fields. The upper panels show magnetization in a larger scale for better visibility. (**a**) O-Sn, (**b**) O-In, (**c**) O-Hg.

**Figure 3:**
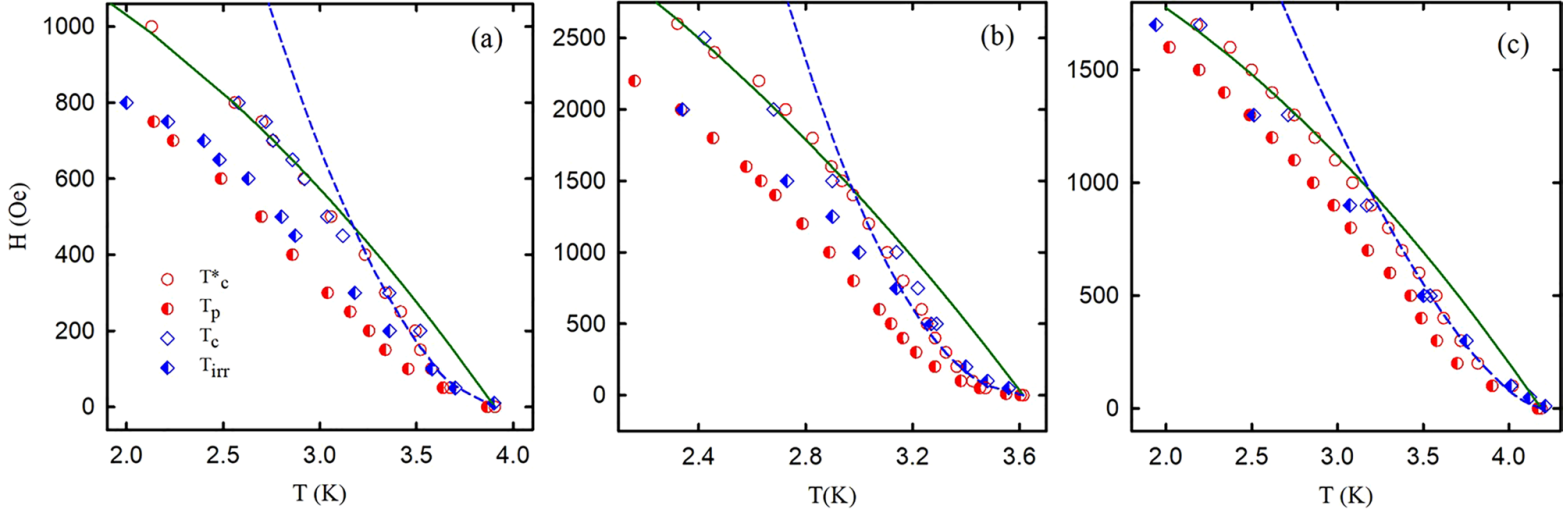
Phase diagrams of opal nanocomposites. The $T_{c}^{*}$, $T_{p}$, $T_{c}$ and $T_{irr}$ notations are defined in the text. The solid and dashed lines are fits using the two liquid model and power law functions. Panels (**a**)–(**c**) correspond to O-Sn, O-In, and O-Hg, respectively. Error of data is less than the symbol size.

Figure 2a–c shows temperature dependences of the dc magnetization under different magnetic fields for O-Sn, O-In, and O-Hg. The nanocomposites demonstrate pronounced diamagnetism below *T*_*c*_ with narrow transitions at low fields. Increasing magnetic field not only depresses *T*_*c*_ but also broadens the transition. The highlights of dc magnetization measurements are huge differences between zero-field cooling (ZFC) and field cooling (FC) magnetizations near 2 K (almost three orders in value) and splitting of ZFC and FC curves immediately at the onset of superconductivity at low external magnetic fields. At higher external magnetic fields, the difference between ZFC and FC magnetizations becomes much smaller, and a part of ZFC magnetization overlaps with FC magnetization near *T*_*c*_. In addition, we observed the pronounced paramagnetic Meissner effect in O-In and O-Hg. Our previous studies revealed a similar behavior^21^ for other confinement with much narrower pores. To determine *T*_*c*_ from the dc magnetization, we choose the onset point of diamagnetism from the ZFC curves. The irreversibility temperatures (*T*_*irr*_) were determined by the splitting of the ZFC and FC curves. The obtained *T*_*c*_ and *T*_*irr*_ are shown in Fig. 3. Notably, O-Sn has the highest filling factor (see “Methods”) leading to the narrowest superconducting transition width among the samples.

### ac susceptibility

**Figure 4:**
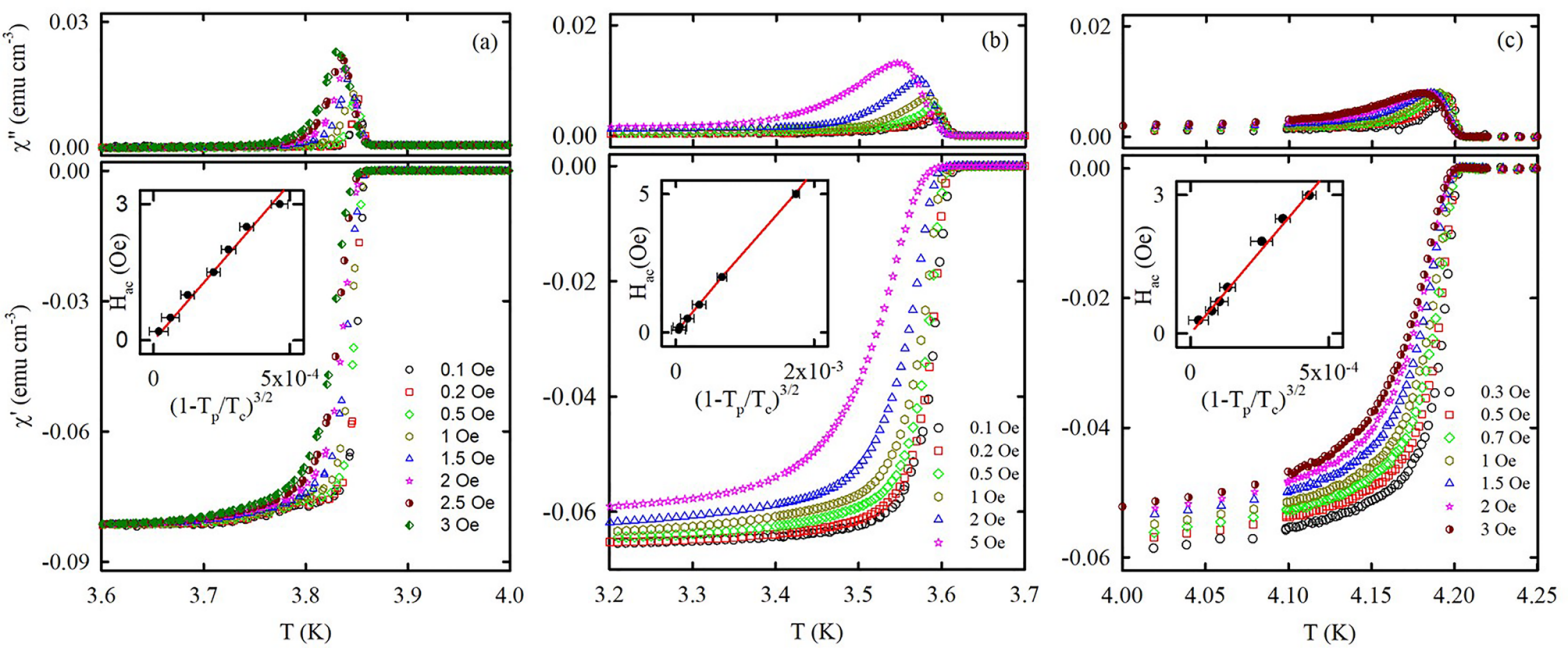
Temperature dependences of ac susceptibility at zero field for (**a**) O-Sn, (**b**) O-In, and (**c**) O-Hg with different ac driving amplitudes indicated in the lower panels. The ac driving frequency is 500 Hz. The upper and lower panels show the imaginary and real parts of ac susceptibility, respectively. The insets show the dependences of the ac field amplitudes on (1 − *T*_*p*_/*T*_*c*_)^3/2^. The solid lines are linear fits.

Figure 4a–c presents the temperature dependences of ac susceptibility for the O-Sn, O-In, and O-Hg samples, respectively, under different ac driving amplitudes *H*_*ac*_ at zero bias field. At *H*_*ac*_ = 0.1 Oe the real part of ac susceptibility *χ*′ shows a sharp diamagnetic shielding and the imaginary part *χ*″ shows a quite narrow peak associated with the superconducting transition. Increasing the ac driving field noticeably broadens the superconducting transition, slightly reduces the onset of superconductivity and strongly shifts the peak maximum *T*_*p*_ to lower temperatures. We use the least squares polynomial fitting to determine *T*_*p*_ for every *H*_*ac*_. The insets in Fig. 4 show that the relation of *T*_*p*_ with *H*_*ac*_ follows *H*_*ac*_ ~ (1 − *T*_*p*_/*T*_*c*_)^3/2^ for all our nanocomposites.

To measure the ac susceptibility at different frequencies and bias magnetic fields, we fix the ac driving amplitude at 1 Oe because it provides a good signal to noise ratio. The applied frequencies cover three orders of magnitude. This allows us to calculate the vortex activation barrier. The temperature dependences of the ac susceptibility at different frequencies and 500 Oe external magnetic field are shown in Supplementary Fig. S1 as an example. Similar results were obtained in other bias fields. Although the temperature variations of *χ*″ and *χ*′ depend feebly on frequency, *T*_*p*_ increases regularly with increasing the ac driving frequency.

**Figure 5:**
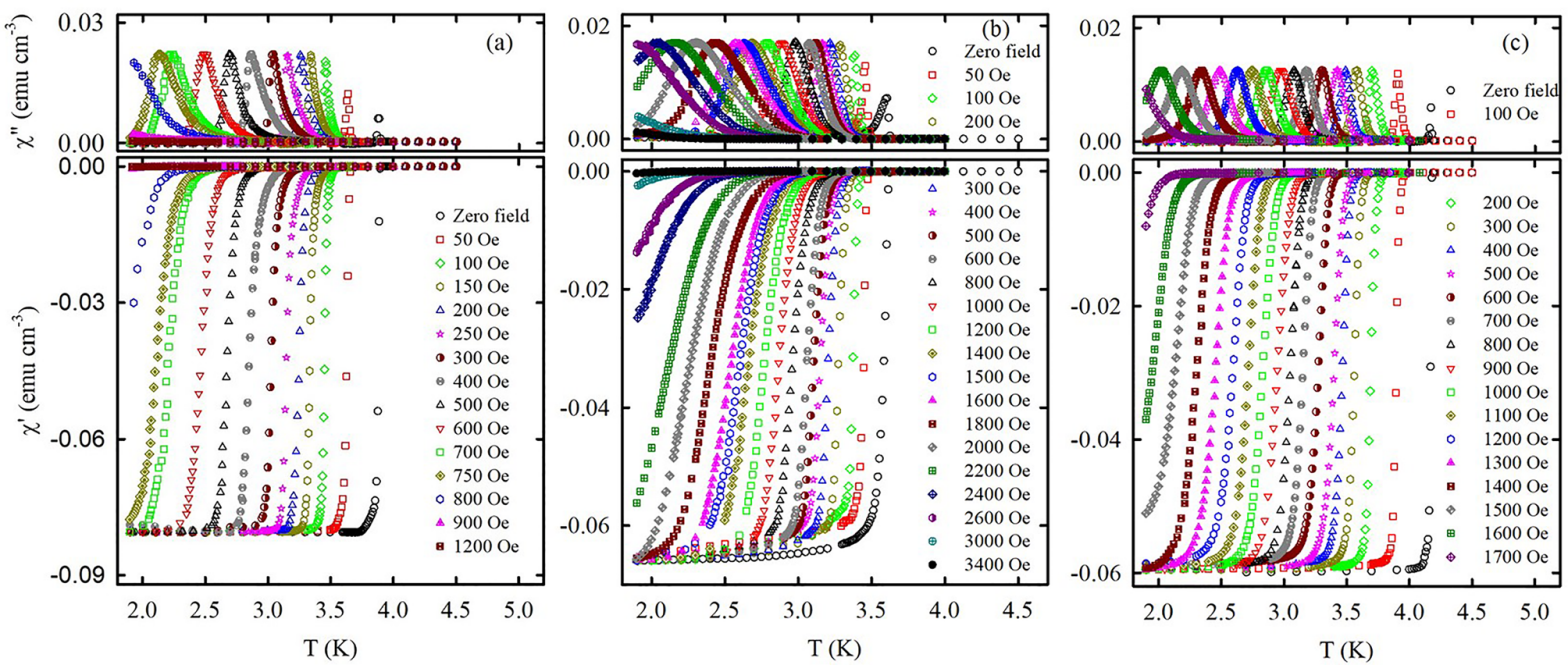
Temperature dependences of ac susceptibility for (**a**) O-Sn, (**b**) O-In, and (**c**) O-Hg under various bias magnetic fields indicated in the lower panels. The lower and upper panels show *χ*′ and *χ*″, respectively. The ac frequency and ac field amplitude are 500 Hz and 1 Oe, respectively.

Figure 5a–c shows the representative temperature dependences of *χ*′ and *χ*″ at various external magnetic fields for the 500 Hz ac driving frequency. The plots of *χ*′ allow us to evaluate the onset temperature of superconductivity $T_{c}^{*}$ at various bias fields. $T_{c}^{*}$ and $T_{p}$ are shown in the phase diagrams in Fig. 3. Notably, the $T_{p}$ values correspond to the ac frequency 0.5 kHz.

### SANS

The SANS results were obtained in the q-range between 0.06 and 0.4 Å^−1^ at 0.5 and 5 K (well below and above the phase transition temperatures, respectively) and in a magnetic field of 400 Oe. The SANS patterns were typical for an FCC packed opal matrix. To get information about the vortex phase, we compared the SANS results obtained at the two temperatures (0.5 K and 5 K, below and above T_c_). The difference between the patterns revealed only irregular scattering within the noise level that denied the existence of any more or less regular vortex structure. We show the SANS results in Supplementary Fig. S2. Results at different magnetic fields also suggest a highly disorder vortex structure.

## Discussion

The results shown in Figs. 2, 4, 5 and Supplementary Fig. S1 attribute the loaded opals to type-II superconductors. As bulk Sn, In, and Hg metals are type-I superconductors, these results confirm the change of type of superconductivity when we confine type-I superconductors to nanopores. This agrees with known studies suggesting that type-I superconductors show type-II behavior when their sizes shrink down to submicron and shorter region owing to mesoscopic effects^22–26^. The general superconducting properties of porous templates loaded with metals can be described by a model of grains linked via superconductor-normal metal-superconductor (SNS) or superconductor-insulator-superconductor (SIS) links^9^. The grains with dendritic morphology consist of several filled neighbor pores which are connected by filled channels^17,18^. The porous volume of empty opal, in principle, is ca. 25% of the total volume. Consequently, the superconducting fraction in the nanocomposite are around 23.8%, 15% and 15% of total sample volume for O-Sn, O-In, and O-Hg. However, all samples show $- 4\pi \chi^{\prime} = 1\sim 0.67$ at the lowest temperature and low field region (see, for instance, Figs. 3 and 4). It illustrates that the linked dendritic grains provide very strong diamagnetic shielding to the whole sample even with more than three-fourth volume occupied by silica spheres and empty pores.

On the other hand, the superconducting transitions for all our nanocomposites are sharp (the transition width is less than 0.5 K at zero field) and no evidence of the second step in magnetization owing to SIS links is seen, we suggest that SNS links provide the main mechanism of Cooper pair tunneling between grains. The supercurrents then screen the almost whole sample volume in agreement with the strong diamagnetism for our nanocomposites at low fields under ZFC condition (Fig. 1). Notably, the transition widths in our nanocomposites are much narrower than in granular high-temperature superconductors^27,28^ and iron-based superconductors^29,30^.

For further understanding the superconductivity in our nanocomposites, we should look into their phase diagrams in Fig. 3. The $T_{c}$ and $T_{c}^{*}$ temperatures almost coincide with each other and form the upper critical field lines. In the low field region, the upper critical field lines show the strong positive curvature which deviates from predictions of the conventional WHH^31^ or two-fluid models^32^. The curvatures of the upper critical lines change the sign with increasing magnetic field. Figure 3 shows different fitting curves at low and high magnetic fields. The fitting curves at high magnetic fields correspond to the two-fluid model $H_{c2} = H_{c2} \left( 0 \right)\left[ {1 - \left( {\frac{T}{{T_{c} }}} \right)^{2} } \right]$. At low magnetic fields, the upper critical field curves with positive curvature can be fitted by a power law $H_{c2} = H_{0} \left[ {1 - \left( {\frac{T}{{T_{c} }}} \right)} \right]^{n}$, where *n* equals 2 for O-In and 3/2 for O-Sn and O-Hg. This allows us to define the mean crossover fields $H_{cr}$ at the interceptions of these fitting curves. They are about 480, 1440, and 960 Oe for O-Sn, O-In, and O-Hg, respectively. Our results show similar dependences for some unconventional superconductors^33–35^.

The approximations made for high magnetic fields using the two-fluid model give us the values of $H_{c2} \left( 0 \right)$ listed in Table 1. Table 1 shows the remarkable enhancement of $H_{c2} \left( 0 \right)$ compared to the critical fields in the relevant bulk metals^36,37^. Within the framework of the Ginzburg–Landau theory, the upper critical field relates to the coherence length:

1 $$\xi \left( 0 \right) = \sqrt {\frac{{\Phi_{0} }}{{2\pi H_{c2} \left( 0 \right)}}} ,$$

where $\Phi_{0}$ is the flux quantum. The coherence lengths calculated are also presented in Table 1. Their values are of the same order as the pore sizes of the opal template which are less than 67.5 nm (0.225 *D*) for tetrahedral pores and 124.2 nm (0.414 *D*) for octahedral pores, where *D* is the silica sphere size (in this study, *D* = 300 nm, see “Methods”). This suggests that the electron scattering in metals confined to nanopores is the main cause of the coherence length reduction.

**Table 1:** Critical fields *H*_*c*_(0) for bulk metals used in the nanocomposites, calculated upper critical fields *H*_*c*2_(0), crossover fields $H_{cr}^{*}$ on the activation barrier lines, ratio *H*_*c*2_(0)/*H*_*c*_(0), and calculated correlation length *ξ* for the nanocomposites studied.

	O-Sn	O-In	O-Hg
*H*_*c*_(0)	285.7 ± 0.5 Oe^36^	308.7 ± 0.5 Oe^36^	415.1 ± 0.5 Oe^37^
*H*_*c*2_(0)	1400 ± 40 Oe	4470 ± 90 Oe	2300 ± 40 Oe
$H_{cr}^{*}$	~ 0.5 kOe	~ 1.5 kOe	~ 1.0 kOe
*H*_*c*2_(0)/*H*_*c*_(0)	~ 5	~ 15	~ 5
*ξ*	54 ± 2 nm	28.0 ± 1.5 nm	39.0 ± 1.0 nm

The phase diagrams in Fig. 2 comprise also $T_{irr}$ versus field lines that closely reproduce the anomalous shape of the upper critical field lines with the positive curvature at low magnetic fields. Following the definition of $T_{irr}$^28,38^, the $T_{irr}$ lines separate the immobile and mobile vortex phases. The irreversibility lines almost coincide with the upper critical field lines at very low fields suggesting that vortices are strongly pinned immediately below *T*_*c*_ with a glassy disorder structure. At higher fields, the irreversibility lines are far from the upper critical field lines indicating an unpinned state between them and show common behavior.

Figure 4 and Supplementary Fig. S1 show the typical type-II behavior with thermally assisted vortex motion which conforms with ideas developed in^39,40^. The feeble variations of the ac susceptibility with frequency mean that the critical current $J_{c}$ weakly depends on voltage. Consequently, the critical current density is proportional to the amplitude *H*_*ac*_ of the ac field at the peak temperature *T*_*p*_. The insets to Fig. 4 show that the variations of *T*_*p*_ with *H*_*ac*_ follow a power-law function *H*_*ac*_ $\propto$(1 − *T*_*p*_/*T*_*c*_)^3/2^ which indicates the same dependence of the critical current. This also agrees with our modeling the nanocomposites linked by SNS junctions.

**Figure 6:**
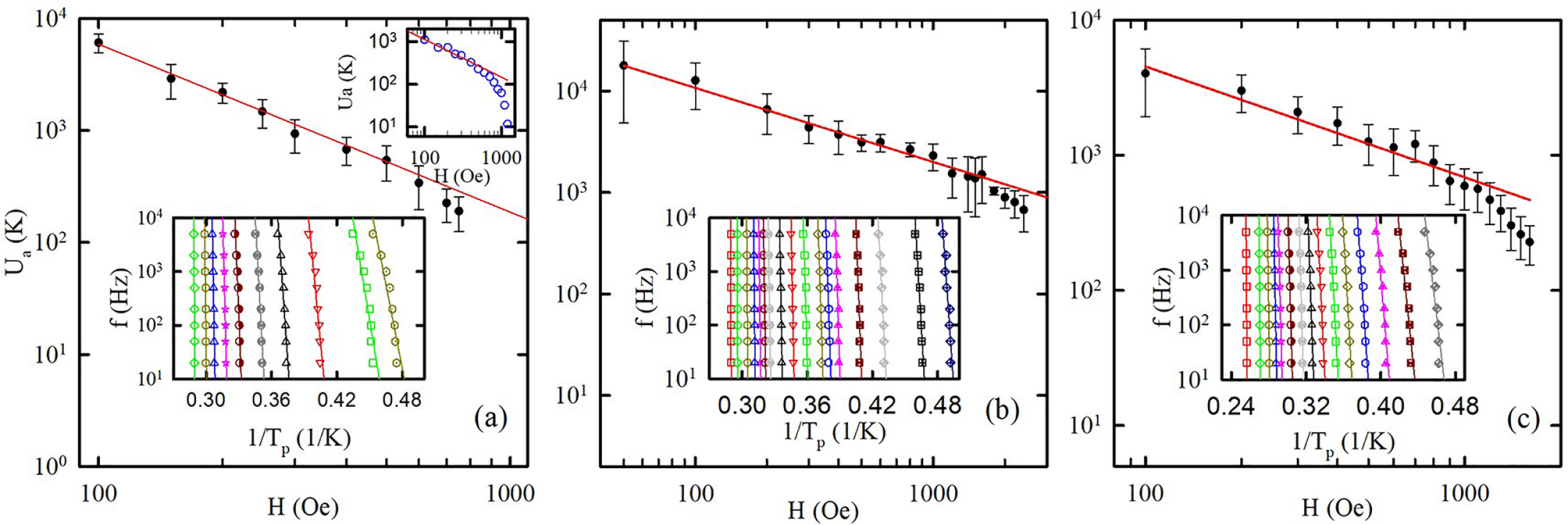
The bias magnetic field versus calculated activation barrier for (**a**) O-Sn, (**b**) O-In, and (**c**) O-Hg. The red straight lines are power law fits as discussed in the text. The insets show the Arrhenius plots for *T*_*p*_ with linear fits. The upper inset in the panel (**a**) shows the variations of activation barriers with field obtained from resistance measurements for O-Sn.

Following the treatment for different kinds of superconductors^9,41–43^ the ac susceptibility data gives us the vortex activation barrier $U_{a}$ within the framework of the thermally activated vortex motion model. The insets to Fig. 5 demonstrates the validity of the Arrhenius plots for $T_{p}$ in the opal based nanocomposites while Fig. 5 itself shows the field dependence of the activation barriers. Notably, the superconducting transitions are too sharp to find the reliable vortex activation barriers for applied fields less than 100 Oe. Figure 6 shows the power-law field dependence *U*_*a*_(*H*) ∝ *H*^−*α*^ of the activation barrier at lower fields for all the nanocomposites. The exponent *α* is equal to 1.5, 0.73, and 0.82 for O-Sn, O-In, and O-Hg, respectively. For O-In and O-Hg a kink emerges at higher fields. We define the crossover fields $H_{cr}^{*}$ as fields at which the $U_{a} \left( H \right)$ curves deviate from their original flat slope in O-In and O-Hg. The values of $H_{cr}^{*}$ are equal to 1.5 and 1.0 kOe for O-In and O-Hg, respectively. For O-Sn the occurrence of the kink is unjustified while the trend to a kink is seen near 0.5 kOe. To clear this situation up, we measured resistance at different fields for the O-Sn sample. Palstra et al.^44^ proposed a model to describe the temperature dependence of the resistance influenced by vortex motion in the mixed state which follows the Arrhenius law:

2 $$R = R_{n} \exp \left( { - \frac{{U_{a} }}{T}} \right),$$

where $R_{n}$ is a resistance in the normal state. The upper inset to Fig. 6a shows the activation barriers found from measurements of resistance at various magnetic fields. One can clear see a kink at a field $H_{cr}^{*}$ of ca. 0.5 kOe. The field dependence of the activation barrier becomes much stronger above this kink. The field 0.5 kOe is just the field at which the trend to a kink is noticeable in Fig. 6a.

**Figure 7:**
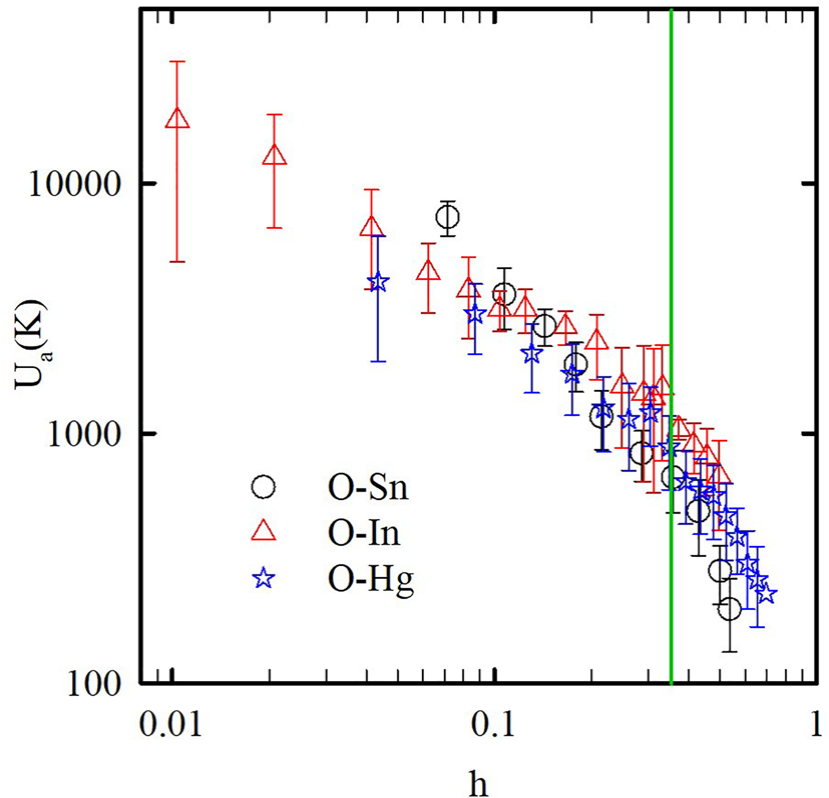
Dependences of activation barriers on normalized fields for O-Sn, O-In, and O-Hg. The solid line is a mark at *h* = 0.35.

**Figure 8:**
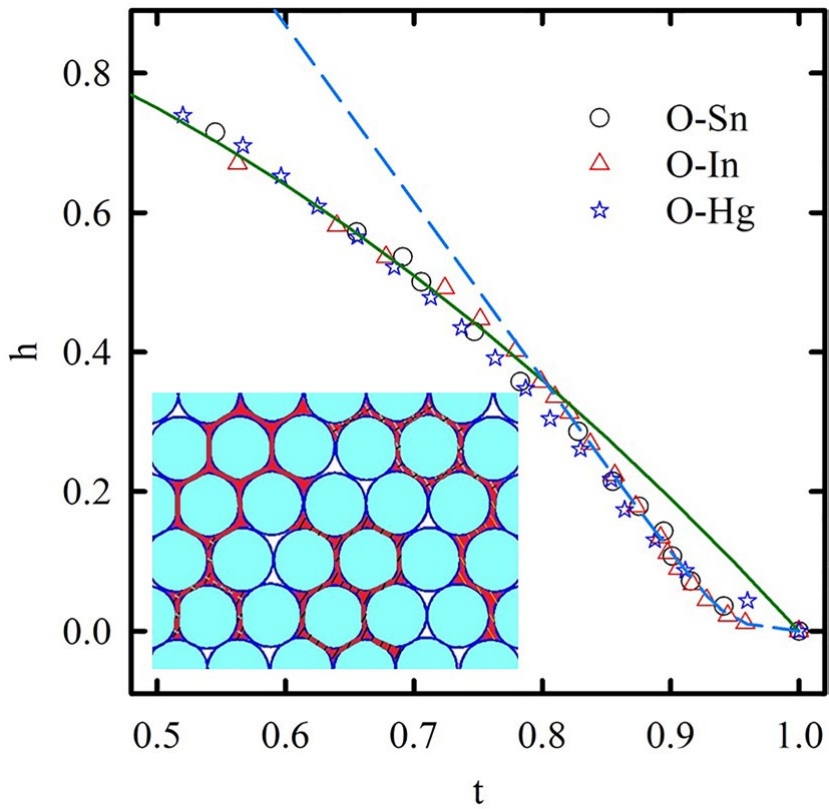
The normalized phase diagram of O-Sn, O-In, and O-Hg. The green solid line shows the fitting curve of two-fluid model. The blue dashed line represents the proximity effect model: $1 - t = \alpha h + \frac{\lambda \beta h}{{2\lambda + \beta h}}$, with fitting parameters: $\alpha = 0.394$, $\beta = 0.058$, and $\lambda = 19.48$. The inset presents a cartoon cross-section view of the opal nanocomposites. The blue balls show the opal template and the red color indicates metallic inclusions forming the dendritic grains. Different single crystalline grains are marked by different hatching.

To emphasize the common features in the superconductivity of the three nanocomposites with different metals but similar morphology, we plotted the dependences of the activation barriers on the reduced field $h = \frac{H}{{H_{c2} \left( 0 \right)}}$ using the data from Fig. 6 to a single plot in Fig. 7. The dependences of the activation barriers on the reduced field are very similar for these nanocomposites with a common kink at $h_{cr}^{*} \approx$ 0.35. To compare it with the phase diagrams, the upper critical field lines scaled with respect to the reduced field $h$ and reduced temperature $t = \frac{T}{{T_{c} }}$ for the three nanocomposites are shown in Fig. 8. They also demonstrate a common behavior with *h*_*cr*_ $\approx$ 0.35.

The change in the field dependence of the activation barriers for different kinds of superconductors is known to correlate with a transformation in the vortex system^41–43^. The fact that, for our nanocomposites, this transformation happens at a field *h*_*cr*_ similar to $h_{cr}^{*}$ allows us to suggest that the crossover in the upper critical field line also correlates to the vortex system transformation.

To understand qualitatively how the configuration of the metallic network within the opal matrix can influence the H_c2_(T) and U_a_(H) lines and produce crossovers on these lines at a similar normalized field, we combine the ideas of the Josephson linked grains^45^ and the surface barriers^46–48^ of grains.

At low magnetic fields, the grains linked by SNS junctions behave like a bulk type-II dirty superconductor. Grain boundaries and silica spheres pin down vortices as the surface barriers preventing the vortices to enter into grains if the external magnetic field is small. To move from a pinning center to another one, the vortices need to overcome high potential barriers corresponding to the weak field parts of the curves in Fig. 5. With increasing the magnetic field, it gradually breaks the links between grains. The proximity effect disappears and the coherence length decreases giving a higher upper critical field at low temperatures (Fig. 3). The upper critical line shows a crossover from the positive to negative curvature at a field, which breaks the intergrain links. When the intergrain space is in the normal state, the vortices enter the grains, and their mobility depends on smaller activation barriers giving a kink on their variation with the magnetic field (Figs. 6, 7). Quantitatively, the H_c2_(T) line in the range of the positive curvature allows treatment within a model suggested in^49^. The model considers a stack of irregular superconducting and nonsuperconducting layers coupled with Josephson links. The Josephson coupling makes the order parameter nonzero on the nonsuperconducting layers through a proximity effect. The spatial variation of the order parameter gives rise to the positive curvature close to T_c_. At high magnetic fields, the order parameter on the nonsuperconducting boundaries becomes negligible as the correlation length decreases. Considering the dendritic grains within opal matrices, crystalline boundaries serve as SNS Josephson links (see the inset to Fig. 8). Then, for our quasi-one-dimensional superconducting networks, we can use the relationship (13) from^49^ to fit the experimental dependences of the upper critical field on temperature. The obtained fitting curve is shown in Fig. 8 also giving us similar reduced crossover field *h*_*cr*_ $\approx$ 0.35.

Owing to the complex structure of the opal-based nanocomposites, we may expect highly disorder vortex phases in our samples. It explains why we are unable to observe any evidence of the vortex lattice state for the O-Sn nanocomposite by SANS. It means that the kink corresponds to the field-induced vortex system transformation between two highly disordered structures.

The coincidence of the reduced magnetic field $h_{cr}^{*}$ of the kink on the field dependences of the potential barriers with the crossover *h*_*cr*_ in the upper critical field line for the three nanocomposites emphasizes the role of the metallic network morphology and novel vortex dynamic governed by the geometry of the opal template. As we mentioned above, the positive curvature of the upper critical field line and the abnormal field variation of potential barriers can be observed in some unconventional superconductors. These anomalies are often very similar to those found here for our nanocomposites. This allows us to suggest a possible common nature of these anomalies caused by a dendritic second phase, which needs new models to understand its behavior.

In conclusion, measurement of the dc and ac magnetizations in opal templates loaded with tin, indium, and mercury revealed common features of the nanocomposites with type-II superconducting behavior. The upper critical field lines show a crossover from the positive to negative curvature with increasing magnetic field. The field dependences of activation barriers have a kink corresponding to the vortex systems transformation. The fields of the curvature crossover and of the kinks are quite similar to each other. Moreover, the normalized phase diagrams and field dependences of activation barriers demonstrate a common transformation in the vortex system at a reduced crossover field *h*_*cr*_ ≈ 0.35 for the three nanocomposites which emphasizes the role of morphology of confined metallic networks inducing an unconventional-like behavior at the low field regime.

## Methods

The opal template was formed by silica spheres with a mean diameter of 300 nm and sintered. The regular structure of tetrahedral and octahedral pores connected with each other is inside of the opal template. The pore to sample volume ratio of the opal is ca. 25%. Liquid metals with purity 99.99% were embedded into the opal matrices by pressure up to 10 kbar at temperatures above the melting points of tin, indium, and mercury. The three metals do not wet the silica surface. The filling factors for the loaded opals were evaluated by weighing the samples before and after their filling. The Sn loaded opal (O-Sn) had the highest filling factor of ca. 95%. The filling factors of O-In and O-Hg were about the same and equal to ca. 60%. Also, we check the opal template and the filling factor near the sample surface by the FEI Helios NanoLab G3 CX DualBeam microscope. The cross-sections were milled by a Ga ion source and, to get a better micrograph, we deposited a thin layer of Pt to prevent scratching.

The dc magnetization of the opal-metal nanocomposites was measured using Quantum Design MPMS 3. The temperature dependences of dc magnetization were monitored with the ZFC and FC protocols under different external magnetic fields. To reduce the residual field below 2 Oe before measurements, we set the magnetic field to a few Teslas and then ramped it down to zero. The changing temperature rate, measurement step, and temperature stabilization time were set to 0.3 K/min, 0.02 K, and 60 s, respectively, in order to prevent temperature from overshooting and compensate thermal lag. For each measurement, the averaging time was 10 s sufficiently when magnetization is higher than 10^–6^ emu. The field response of the superconducting magnet was checked by both Pd and Dy_2_O_3_ samples.

The ac susceptibility was measured using Quantum Design PPMS. Temperature variations of ac susceptibility were monitored with the FC protocol under different bias fields, amplitudes, and frequencies of the ac driving field. The changing temperature rate and stabilization time were set to 0.3 K/min and 120 s, respectively. Close to the superconducting transition, the temperature step was set to 0.01 K or smaller. The ac driving field for measurements at different frequencies was set to 1 Oe. We also checked the field response of the PPMS superconducting magnet with a Toshiba THS 123 Hall sensor.

In addition, we carried out SANS experiments to get information about the vortex system in opal-tin nanocomposite at the SANS-1 instrument operated by TUM and HZG at the Heinz Maier–Leibnitz Zentrum (MLZ) Garching^50,51^. A sample with a volume of ~ 20 mm^3^ was studied. Measurements of FC rocking scans under different magnetic fields, ZFC field scans up to 2 kOe and back to zero were made. The measurements were performed at the base temperature of the ^3^He insert (0.5 K) except for the background measurement (5 K) and with incident beam wavelength of 12 and 9 Å.

## Supplementary Information

Supplementary Information.

## Data Availability

All data measured or analyzed during this study included in this published article and its Supplementary Information are available from the corresponding author on reasonable request.
