# Supplementary material for: The morphologic correlation between vortex transformation and upper critical field line in opal-based nanocomposites
Source: Sci Rep. 2021 Feb 26;11:4807. doi: 10.1038/s41598-021-84343-1 (PMC7910285; doi:10.1038/s41598-021-84343-1)
Supplement: Supplementary file 1 — Supplementary Information. [file 41598_2021_84343_MOESM1_ESM.docx]

The morphologic correlation between vortex transformation and upper critical field line in Opal-based nanocomposites.

M. K. Lee^1*^, E. V. Charnaya^2,3^, S. Mühlbauer^4^, U. Jeng^5^, L. J. Chang^2^, and Yu. A. Kumzerov^6^

*^1^ MOST Instrument Center at NCKU, Tainan 70101, Taiwan*

*^2^ Department of Physics, National Cheng Kung University, Tainan 70101, Taiwan*

*^3^ Department of Physics, St. Petersburg State University, St. Petersburg, Petrodvorets 198504 Russia*

*^4^Heinz Maier-Leibnitz Zentrum (MLZ), Technische Universität München, Lichtenbergstrasse 1, D-85748 Garching, Germany*

*^5^National Synchrotron Radiation Research Center, 101 Hsin-Ann Road, Hsinchu Science Park, Hsinchu 30076, Taiwan*

*^6^ A. F. Ioffe Physico-Technical Institute RAS, St. Petersburg 194021, Russia*

*^*^anion3143@hotmail.com*

Supplementary information.

.


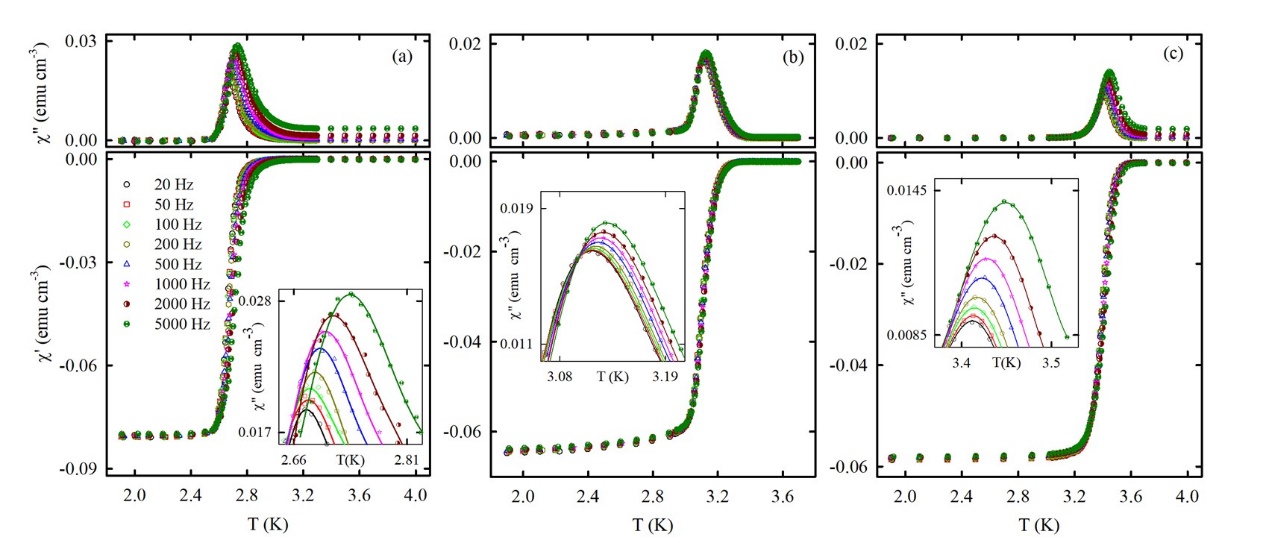


Figure S1. Temperature dependences of ac susceptibility for (a) O-Sn, (b) O-In, and (c) O-Hg at different driving frequencies shown in the lower panels. The ac field amplitude and bias magnetic field are 1 Oe and 500 Oe, respectively. Upper panels: the imaginary part of ac susceptibility; lower panels: the real part of ac susceptibility. The insets show the enlarged ac loss peaks with least square polynomial fitting curves (solid lines).


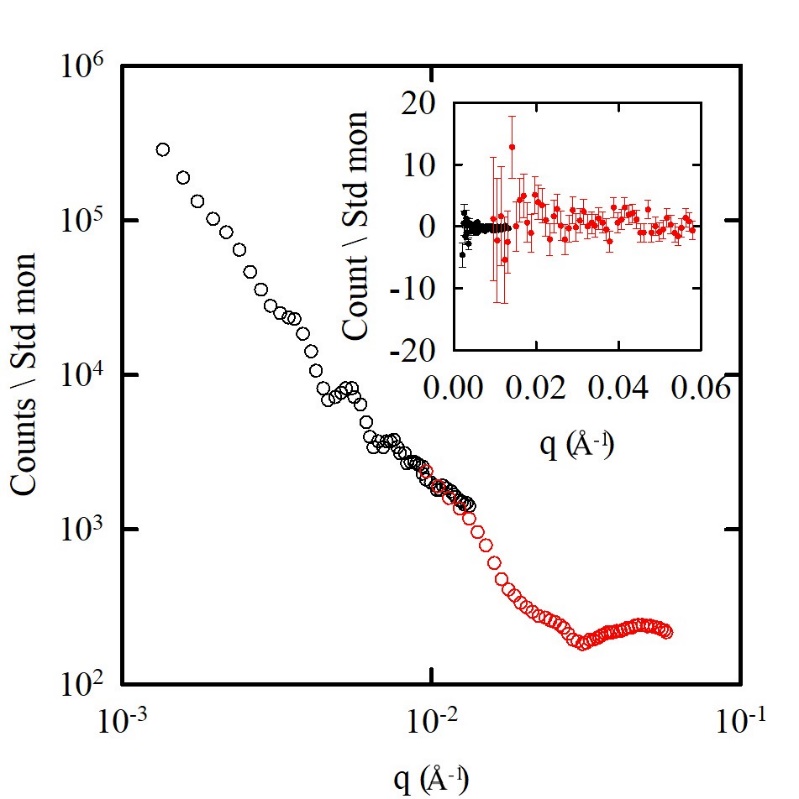


Figure S2. The experimental SANS data on O-Sn. The black and red circles indicate the results measured by incident neutron beam wavelength 9 Å and 12 Å at 0.5 K with a magnetic field 400 Oe. The inset shows the subtraction of results between 0.5 K and 5 K (above Tc). Its values deny any ordered vortex structure at 0.5 K. Our measurements under different fields show similar results
